# Teaching screening, brief intervention, and referral to treatment to social work students

**DOI:** 10.1186/1940-0640-7-S1-A64

**Published:** 2012-10-09

**Authors:** Victoria Osborne, Kalea Benner, Carol Snively, Dan Vinson, Bruce Horwitz

**Affiliations:** 1 School of Social Work, University of Missouri, Columbia, MO, USA; 2 College of Human Environmental Sciences School of Social Work, University of Missouri, Columbia, MO, USA; 3 Family and Community Medicine, University of Missouri, Columbia, MO, USA; 4 Department of Psychiatry, University of Missouri School of Medicine, Columbia, MO, USA

## 

Although social workers encounter many clients with substance use problems, curricula rarely require education on addictions. The screening, brief intervention, and referral to treatment (SBIRT) model was initially directed to practicing physicians. Recently, training has evolved to include medical students. This study describes the first known application of SBIRT training to social work students. The goal was to assess students’ knowledge of and attitudes toward alcohol misuse before and after SBIRT training. Students were given a questionnaire assessing attitudes, knowledge and perceived skills with regard to substance misuse. A computerized training session focused on symptoms of at-risk drinking and implementing SBIRT. Descriptive statistics explained overall knowledge, attitudes, and perceived screening and intervention skills. T-tests compared changes pre- and post-test. Seventy-four social work students (33 undergraduate and 41 graduate) completed the training modules and pre- and post-tests. Significant differences were found in seven of the 13 questions. Students reported more confidence in their ability to assess for alcohol misuse and successfully intervene with clients who have substance use behaviors. They reported feeling more strongly that routine screening and brief intervention were crucial to clinical practice. Incorporating alcohol screening and brief intervention techniques into social work practice is an important aspect of effective treatment. Training students to screen and intervene is critical to improving treatment skills. Teaching SBIRT is a simple and effective way to implement addictions education into social work curricula. Such training appears to increase students’ perceptions of their ability to change client behaviors and reduce client alcohol misuse.

